# Lipoblastome de la fosse sous temporale

**DOI:** 10.11604/pamj.2014.19.323.2623

**Published:** 2014-11-26

**Authors:** Jaafar Najib, Khalid Aniba, Mehdi Laghmari, Mohammed Lmejjati, Houssine Ghannane, Said Ait Benali, Hind Ennadam, Hind Mrabti, Nadia Cherif Idrissi

**Affiliations:** 1 Service de Neurochirurgie, Hôpital Ibn-Tofail, CHU Mohammed VI, Marrakech, Maroc; 2 Service de Radiologie, Hôpital Ibn-Tofail, CHU Mohammed VI, Marrakech, Maroc

**Keywords:** Lipoblastome, fosse infratemporale, IRM, excision totale, Lipoblastoma, infratemporal fossae, MRI, total excision

## Abstract

Le lipoblastome est une tumeur bénigne rare, formée d'adypocytes immatures associés à la présence d'une matrice myxoide, de septas fibreux, et d'une architecture lobulaire. Il survient généralement chez le nourrisson et l'enfant. Cette tumeur touche dans la majorité des cas les tissus sous-cutanés des extrémités et du tronc. Elle est extrèmement rare au niveau de la tète et du cou. Un total de moins de 100 cas a été rapporté précédemment dans la littérature.

## Introduction

Le lipoblastome est une tumeur bénigne rare [1], formée d'adypocytes immatures associés à la présence d'une matrice myxoide, de septas fibreux, et d'une architecture lobulaire. Il survient généralement chez le nourrisson et l'enfant [2]. Cette tumeur touche dans la majorité des cas les tissus sous-cutanés des extrémités et du tronc. Elle est extrêmement rare au niveau de la tête et du cou. Un total de moins de 100 cas ont été rapportés précédemment dans la littérature [3]. Nous rapportant un cas très rare de lipoblastome de la fosse sous temporale.

## Patient et observation

**Figure 1:**
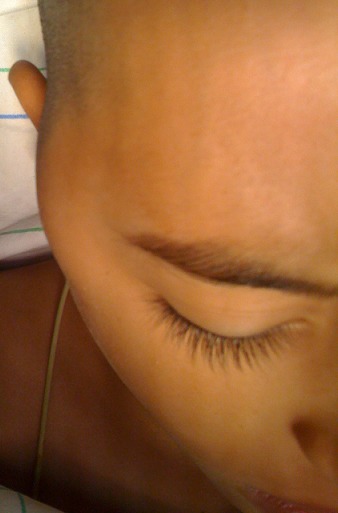
Tuméfaction de la région temporo-mandibulaire droite (flèche)

**Figure 2:**
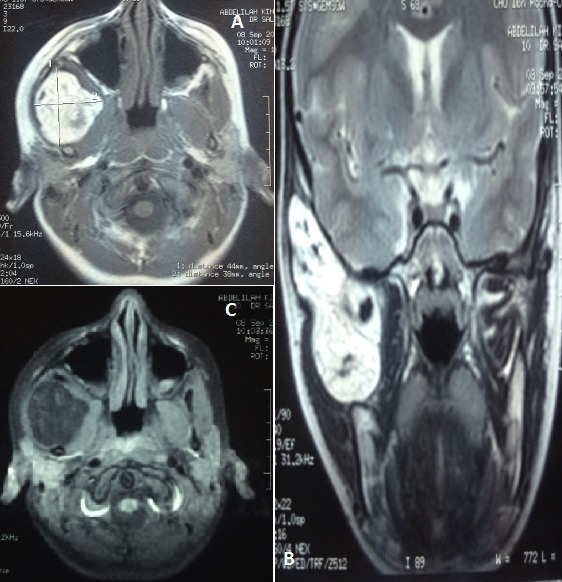
(A) formation de la fosse infratemporale de contours polylobés, bien limitée en hypesignal T1; (B) hypersignal T2 et (C) hyposignal Fat Sat

**Figure 3:**
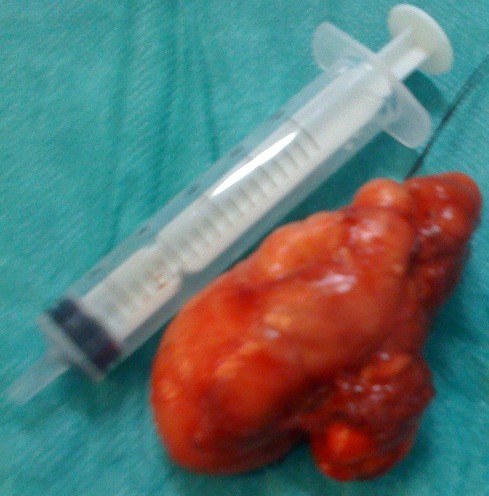
Pièce opératoire

Un enfant de 7 ans, sans antécédents pathologiques, fut hospitalisé pour une tuméfaction temporo-mandibulaire droite augmentant progressivement de volume Figure 1. Cette masse était indolore sans aspect inflammatoire de la peau en regard ni adénopathie satellite. L'imagerie par résonnance magnétique du massif facial montre une masse de la fosse infra-temporale droite à composante graisseuse prédominante avec une composante charnue au centre sans signe d'extension locorégional Figure 2. Le geste chirurgical à consisté en une exérèse totale en masse d'une lésion graisseuse Figure 3 par une voie d'abord temporale droite.

## Discussion

Le lipoblastome est une tumeur bénigne très rare du tissu graisseux embryonnaire [2]. C'est une tumeur qui touche presque exclusivement le nourrisson et l'enfant. L’âge de survenue est inférieur à 3 ans dans 90% des cas et inférieur à un an dans 40% des cas [4]. Bien que rares, les cas survenant chez des adultes ont été rapportés. Carcassone et al a rapporté un cas de lipoblastome du mésentère chez un homme de 63 ans [5]. Stout et Lattes ont également rapportés 6 cas de lipoblastome chez l'adulte [6]. Il existe une prédominance masculine avec un sexe ratio de 3/1 [7].

Le terme de lipoblastome est introduit par Jaffe en 1926 [8]. Chung et Enzingar [9] identifient en 1973 deux formes: Le lipoblastme, tumeur lobulée correspendant à la forme circonscrite de la tumeur et la lipoblastomatose, masse plus profonde, non encapsulée et plus infiltrante à l’égart des structures de voisinage correspondant à la forme diffuse. La majorité de ces tumeurs touchent les tissus sous cutanés des extrémités et du tronc. Elles sont extrêmement rares au niveau de la tête et du cou: Seulement 48 cas ont été signalés dans la littérature anglaise [1]. Habituellement, le lipoblastome est asymptomatique, mais il peut le devenir par sa taille et sa localisation [10]. Rasmussen et al. A mentionné un cas de lipoblastome cervical provoquant une obstruction intermittente des voies respiratoires. San et al a signalé le premier cas d'hémiparésie résultant d'un lipoblastome supraclaviculaire par compression de la moelle épinière [3].

L'apport de l'imagerie est capital dans l'approche diagnostique de ces tumeurs [11]. La radiographie standard, de nos jours presque abondonnée révèle une masse opaque parfois d'allure graisseuse sans calcification. L’échographie montre soit une masse hyperéchogène soit une masse hétérogène contenant des plages hyperéchogènes dues au tissu adipeux, et d'autres faiblement échogènes au niveau des tissus myxoides. Elle est parcourue par des septas. Parfois la tumeur renferme des petites zones hypoéchogènes ou kystiques. [11] Le doppler couleur objective une hypervascularisation uniquement septale. L'IRM, par son caractère non invasif et non irradiant, est souvent préférée à la tomodensitométrie. Elle confirme la nature graisseuse de la tumeur et l'aspect hétérogène en rapport avec le rehaussement des septas fibrovasculaires [2]. Elle permet aussi l'analyse dans les trois plans de l'espace des relations de la tumeur avec les organes adjacents et les structures vasculo-nerveuses. Le lipoblastome présente un aspect aspect bien limité avec hypersignal T1, signal intérmediaire ou hypersignal T2 et une annulation de signal sur les séquences de supression de graisse [7].

Le diagnostic différentiel se pose avec: le liposarcome dans sa variété myxoide au pronostic très péjoratif [12], encore plus rare que le lipoblastome. Il est caractérisé sur le plan histologique par la présence de mitoses anormales [13]; le lipome qui n'existe théoriquement pas chez l'enfant; l'hibernome: tumeur graisseuse bénigne touchant surtout l'adulte jeune, atteignant électivement la cuisse dont la pathogènie et le potentiel de malignité sont encore mal connus [14]. L'aspect en imagerie de ces tumeurs est aspécifique. L'histologie offre une approche plus précise du diagnostic. La mise en évidence de lipoblastes lobulés et la rareté des mitoses fait évoquer le diagnostic de lipoblastome [15]. L'absence de mitoses anormales écarte le diagnostic de liposarcome. L'hibernome [14] possède des caractéristiques cytologiques pathognomoniques (cellules graisseuses matures marron, uniforme à petit cytoplasme). L’étude cytogénétique constitue un complément d'information à l'analyse morphologique particulièrement utile pour le diagnostic de lipoblastome [16]. Elle objective souvent des remaniements caryotipiques portant sur le chromosome 8 (cassure au niveau du bras long) [12].

Le traitement de choix du lipoblastome consiste en une résection chirurgicale complète [17]. Le taux de récidive locale est de 14 à 25% surtout lorsque la résection est incomplète [18]. Une résolution spontanée d'un lipoblastome de la tête du fémur après un an d'observation, a été rapportée [10]. Collins et al a rapporté le cas d'une maturation d'un lipoblastome cervical en un lipome sur rechute de 4 ans après l'exérèse initiale [19].

## Conclusion

Le lipoblastome est une tumeur bénigne très rare du tissu graisseux embryonnaire. Elle est extrêmement rare au niveau de la tête et du cou. Son diagnostic différentiel se pose avec le liposarcome, le lipome et l´hibernome. L'aspect en imagerie de ces tumeurs est aspécifique. L'histologie Et l’étude cytogénétique offre une approche diagnostic précise. Le traitement de choix du lipoblastome consiste en une résection chirurgicale complète.
